# A naturalistic study of plasma lipid alterations in female patients with anorexia nervosa before and after weight restoration treatment

**DOI:** 10.1186/s40337-024-01055-3

**Published:** 2024-07-02

**Authors:** Alia Arif Hussain, Jessica Carlsson, Erik Lykke Mortensen, Simone Daugaard Hemmingsen, Cynthia M. Bulik, René Klinkby Støving, Jan Magnus Sjögren

**Affiliations:** 1 Eating Disorder Research Unit, Mental Health Center, Ballerup, Copenhagen University Hospital - Mental Health Services of the Capital Region of Denmark, Copenhagen, Denmark; 2 Department of Clinical Medicine, University of Copenhagen, Copenhagen, Denmark; 3 Competence Centre for Transcultural Psychiatry, Mental Health Centre Ballerup, Copenhagen University Hospital - Mental Health Services of the Capital Region of Denmark, Maglevænget 21, 2750 Ballerup, Copenhagen, Denmark; 4 Unit of Medical Psychology, Section of Environmental Health, Department of Public Health, University of Copenhagen, Copenhagen, Denmark; 5 Center for Healthy Aging, University of Copenhagen, Copenhagen, Denmark; 6 Center for Eating Disorders, Odense University Hospital, Odense, Denmark; 7 Research Unit for Medical Endocrinology, Odense University Hospital, Odense, Denmark; 8 Research Unit, Child and Adolescent Psychiatry, Mental Health Services in the Region of Southern Denmark, Odense, Denmark; 9 Department of Clinical Research, University of Southern Denmark, Odense, Denmark; 10 Department of Psychiatry, School of Medicine, University of North Carolina at Chapel Hill, Chapel Hill, NC USA; 11 Department of Medical Epidemiology and Biostatistics, Karolinska Institutet, Stockholm, Sweden; 12 Department of Nutrition, University of North Carolina at Chapel Hill, Chapel Hill, NC USA; 13 Department of Psychiatry, Institute of Clinical Science, Umeå University, Umeå, Sweden

**Keywords:** Anorexia nervosa, Eating disorders, Lipids, Cholesterol, Sex hormones, Estradiol, Testosterone

## Abstract

**Background:**

Plasma lipid concentrations in patients with anorexia nervosa (AN) seem to be altered.

**Methods:**

We conducted a naturalistic study with 75 adult female patients with AN and 26 healthy female controls (HC). We measured plasma lipid profile, sex hormones and used self-report questionnaires at admission and discharge.

**Results:**

Total cholesterol (median (IQR): 4.9 (1.2)) and triglycerides (TG) (1.2 (0.8)) were elevated in AN at admission (BMI 15.3 (3.4)) compared with HC (4.3 (0.7), *p* = 0.003 and 0.9 (0.3), *p* = 0.006) and remained elevated at discharge (BMI 18.9 (2.9)) after weight restoration treatment. Estradiol (0.05 (0.1)) and testosterone (0.5 (0.7)) were lower in AN compared with HC (0.3 (0.3), *p* =  < 0.001 and 0.8 (0.5), *p* = 0.03) and remained low at discharge. There was no change in eating disorder symptoms. Depression symptoms decreased (33 (17) to 30.5 (19), (*p* = 0.007)). Regression analyses showed that illness duration was a predictor of TG, age was a predictor of total cholesterol and LDL, while educational attainment predicted LDL and TG.

**Conclusion:**

Lipid concentrations remained elevated following weight restoration treatment, suggesting an underlying, premorbid dysregulation in the lipid metabolism in AN that persists following weight restoration. Elevated lipid concentrations may be present prior to illness onset in AN.

**Level of evidence: III:**

Evidence obtained from well-designed cohort or case–control analytic studies.

## Abstract (plain language summary)

Fat is essential for the human body. Too much fat in the blood can be a sign of underlying illness including heart disease. This study investigated how plasma lipids (fats) are affected in individuals with anorexia nervosa (AN). We included 75 adult female individuals with AN and 26 healthy female controls, and measured lipids, sex hormones, and used questionnaires upon admission and discharge from treatment. We found that low-weight individuals with AN had higher lipids than the healthy controls, and these lipids remained elevated after weight restoration treatment. Additionally, individuals with AN had lower levels of sex hormones (estradiol and testosterone) at their low weight, and they stayed low even after weight restoration treatment. Eating disorder symptoms remained unchanged, but depression symptoms decreased during treatment. In conclusion, the study suggests that individuals with AN have changes in their lipid metabolism, which persists even after weight restoration treatment. We don’t know the reason behind these elevated lipids, and therefore, this should be investigated further in future study.

## Introduction

Anorexia nervosa (AN) has one of the highest mortality rates of all psychiatric disorders [1]. AN is characterized by restricted food intake, resulting in low body mass index (BMI) [2]. Accompanying symptoms include fear of weight gain, aversion to foods rich in fat and sugar, excessive exercise, distorted body image, and an inability to recognize the seriousness of the low weight.

Evidence for effective treatment strategies is lacking especially in adults [3] and chronicity in AN has been reported to be as high as 33% [4]. Furthermore, as the etiology of AN remains largely unclear, we urgently need a better understanding of the etiology and pathophysiology of AN to identify effective treatments.

A recent genome-wide association study has identified eight risk loci associated with AN risk, and single nucleotide polymorphism based genetic correlations suggest that AN has both psychiatric and metabolic components [5]. Moreover, a significant positive genetic correlation between AN and elevated high-density lipoprotein (HDL) cholesterol has been reported, while there was a negative genetic correlation with fat mass, fat-free mass, BMI, obesity, type 2 diabetes, fasting insulin, insulin resistance, and leptin [5].

Likewise, decades of clinical research have provided evidence for elevated lipid concentrations in the majority of patients with AN, mirroring the recently reported significant genetic correlations [6]. However, only few longitudinal studies measure lipid concentrations during weight restoration. These studies included small samples and reported conflicting results. Some studies found normalized lipid concentrations following (partial) weight restoration, whereas others found persistently elevated concentrations [7–17]. Follow-up was 1–14 months across these longitudinal studies and two studies investigated lipids in fully weight recovered patients with sample size n = 21 [17] and n = 5 [12], respectively. Matzkin et al. included a 4-month follow-up (median BMI increased from 18 to 20 kg/m^2^) and found significantly elevated total cholesterol at baseline in AN compared with healthy control participants (HC), and total cholesterol non-significantly decreased at follow-up in the AN group. Mordasini et al. followed-up the patients with AN after 14 months with BMI increasing from 13.1 to “original weight” without further specifications on the follow-up BMI. Likewise, this study found significantly elevated cholesterol concentrations in the AN group at baseline compared with HC; however, after weight restoration, cholesterol concentrations normalized/decreased again.

The high mortality rates for AN are related to both severe somatic complications and suicidality [1, 18]. Altered lipid concentrations have also been reported in individuals with higher suicidality risk in both AN [19] and in other psychiatric illnesses [20] which, furthermore, could be influenced by the changes in serotonin system functionality found in individuals with higher suicidality risk and in victims of suicide [21].

Numerous other endocrine and metabolic changes have been reported in low-weight individuals with AN, and most of these changes seem to be adaptive in the state of malnutrition and underweight [22]. These alterations include amenorrhea and sex hormone changes. It has been suggested that irregularities in the menstrual cycle could be explained by changes in lipid concentrations affecting metabolism and steroid hormones, i.e. estradiol, a precursor to cholesterol [23]. Blood estradiol concentrations are usually decreased in AN. Endogenous estradiol appears to be cardioprotective, and studies of postmenopausal estradiol deficiency [24] show associations with adverse changes in metabolic risk factors [25]. A small (n = 18) cross-sectional study in individuals with AN also observed a shift to more atherogenic lipoprotein subclasses [26]. Furthermore, a recent lipidomic study investigating adolescents with AN before and after weight restoration (from BMI 15 to 19.5) points towards lipid dysregulation with similarities to obesity and other features of the metabolic syndrome despite the low weight of the patients with AN [27]. A similar investigation of the lipidome in an adult population with AN pointed in the same direction [28]. Persistently elevated lipid concentrations were reported in a study investigating long-term outcomes from a 10-year follow-up of women living with AN of the restrictive subtype according to The Diagnostic and Statistical Manual of Mental Disorders 5th edition (DSM-5) [29], although longitudinal studies with larger sample sizes would improve the level of evidence for this finding.

The longitudinal investigation of lipid concentrations and sex hormones in AN could reveal the underlying pathophysiology by determining whether the observed alteration in lipid concentrations is a merely a consequence of starvation (state-related), or part of an actual premorbid illness mechanism /trait-related). Further clarification of the role of lipids and sex hormones in AN in large longitudinal studies could help identify mechanisms underlying AN, and ultimately, lead to better treatment options and decrease in mortality.

We hypothesized, that (1) plasma lipid concentrations would be elevated in patients with AN pre-treatment compared with HC, whereas sex hormones would be lower; (2) plasma lipid concentrations would remain elevated after weight restoration treatment (measured at discharge); (3) plasma lipids concentrations would be higher in patients with longer illness duration and with severe eating disorder and depressive symptoms.

Therefore, the objectives of the present naturalistic study were to (1) compare plasma lipid and sex hormone concentrations in individuals with AN and in healthy controls; (2) compare plasma lipid and sex hormone concentrations before and after weight restoration treatment in individuals with AN; (3) explore age, BMI, AN subtype, illness duration, education level, and eating disorder and depression symptoms as predictors of lipid concentration in individuals with AN.

## Materials and methods

This study is part of the naturalistic PROspective Longitudinal all-comers inclusion study in Eating Disorders (PROLED) [30] at Mental Health Centre Ballerup, Denmark, and includes patients and controls enrolled between 2016–2020. ClinicalTrials.gov Identifier: NCT03224091. All patients attending a pre-treatment assessment for eating disorders at Mental Health Centre Ballerup are offered to participate in the PROLED-study if they fulfill the inclusion and exclusion criteria.

The recruitment of patients with eating disorders for the overall PROLED-study started the 10th of January 2016 and is planned to continue for 10 years. The PROLED inclusion criteria for patients are: Eating disorder diagnosis according to DSM-5 (AN, bulimia nervosa, binge-eating disorder, avoidant/restrictive food intake disorder (ARFID), and eating disorder not otherwise specified), female or male sex assigned at birth, and ages between 18–65 years. Exclusion criteria are: Involuntary treatment. Patients were recruited from the in- and outpatient departments and day hospital for eating disorders.

The inclusion criteria for HC are: Female or male sex assigned at birth, BMI in the normal range, and ages between 18–65 years. Exclusion criteria: Any sign of a disorder, mental or physical, as judged by the physician after a complete health investigation. Healthy volunteers were recruited from advertisements in newspapers and from the PROLED website.

Pregnancy and lactation are not exclusion criteria, as no experimental medicine is used in the PROLED-study.

This study was performed in accordance with the Declaration of Helsinki and was approved by the Ethics Committee of Central Region in Copenhagen. All participants signed informed consent prior to the study.

### Participants in the present study

**Fig. 1:**
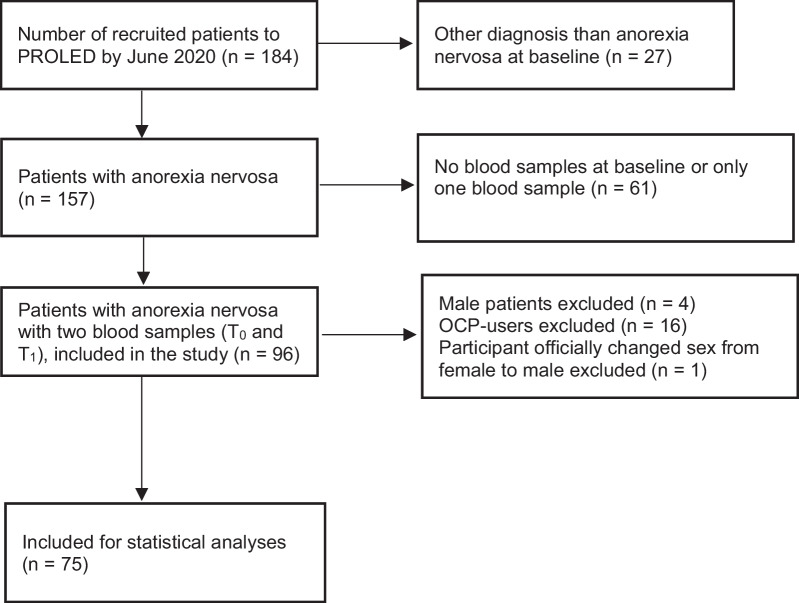
Flowchart over inclusion for the present study. PROLED, PROspective Longitudinal all-comers inclusion study in eating disorders; n, number of participants; OCP, oral contraceptive pills

The current study is a substudy of the PROLED-study, and therefore all participants fulfilled the inclusion criteria and did not meet the exclusion criteria for participation in the PROLED-study. Furthermore, for the present study we only included female patients with plasma blood samples at two time points i.e., at baseline (T_0_) and after weight restoration treatment (T_1_). Seventy-five female individuals with AN (median age 24 years, baseline BMI 15.3 kg/m^2^) and 26 healthy female control participants (HC) (median age 28 years, BMI 22.4 kg/m^2^) were included in the present study. We also excluded participants using oral contraceptive pills as their plasma lipid concentrations could be affected [31]. Inclusion flowchart is presented in Fig. 1. The patients met the diagnostic criteria in DSM-5 for AN [2] and were further divided into the restricting AN and binge-eating/purging AN subtypes. Diagnosis and clinical evaluation of all referred and included patients at baseline were performed by an experienced clinician with the semi-structured interview Eating Disorder Examination (EDE). Furthermore, psychometric self-report questionnaires were completed by the included patients.

The HC were included in the study to assess whether the measured biomarkers and clinical findings differed from the included patients with AN at baseline. The HC were examined identically to the patients at baseline by an experienced clinician. Assessments included screening with Structured Clinical Interview for DSM Disorders (SCID), questions about mental illnesses in the past 5 years, and the psychometric questionnaires.

The psychometric self-report questionnaires were completed at the time of blood samples at T_0_ and again at T_1_. Questionnaires were e-mailed via Research Electronic Data Capture (REDCap; [32]) hosted at www.redcap.regionh.dk.

For the majority of patients, blood was drawn at the day of discharge or a few days before. In the case of premature discharge, the last weekly blood sample was used for the follow-up/T_1_ timepoint analyses and questionnaires were sent electronically through the secure platform digital postbox e-Boks used by public authorities.

### Anthropometric examination and blood sampling

All participants with AN and HC were examined at baseline upon entering the PROLED-study, the AN group after basic stabilizing treatment including prevention of the refeeding syndrome. Every patient was individually monitored for the refeeding syndrome at the beginning of treatment and until they were somatically stable with acceptable blood electrolyte concentrations and without objective signs of the refeeding syndrome (usually within a week). The initial renourishment schedule was based on the patient’s caloric intake prior to treatment i.e., starting low, if the patient had a very low calorie intake. Once the patient was medically stable, they proceeded to a weight restoration renourishment schedule adjusted to meet the weight restoration requirement of 1 kg/week with the target BMI 20 kg/m^2^. The patients were prescribed five supervised daily meals (three main meals and two snack meals). In the case of somatic complications, the patient was transferred to the Department of Endocrinology at Herlev Hospital, Denmark, and returned to the Department of Eating Disorders, once they were somatically stable.

Anthropometric examination and blood sampling were repeated when the participants with AN were discharged. For all participants, weight and height were determined by a nurse, research assistant or medical doctor on a calibrated scale without clothes and shoes. BMI was calculated (weight in kilograms divided by height in meters squared: kg/m^2^).

Blood samples for total cholesterol, high-density lipoprotein (HDL), low-density lipoprotein (LDL), very low-density lipoprotein (VLDL), triglycerides (TG), free fatty acids (FFA), sex-hormone binding globulin (SHBG), testosterone, estradiol, progesterone, luteinizing hormone (LH), and follicle-stimulating hormone (FSH) were sampled between 8 a.m. and 12 p.m. Lipid concentrations were measured by enzymatic determination and absorption photometry on Cobas 8000, c702 modul (Roche Diagnostics, 2014, CV_max_ 5%). Sex hormones were measured by electrochemiluminescence immunoassay (ECLIA) on Cobas 8000, e801 module (Roche Diagnostics, 2017, CV_max_ 7%). VLDL was calculated as TG/2.2 (unit: mmol/L). The patients followed their prescribed meal schedules eating 5 times per day. Since this was a naturalistic study and it was regarded as disruptive to treatment goals and potentially triggering of ED behaviors thereby jeopardizing treatment adherence, patients were not fasting at the time of blood draws. However, although fasting lipid concentrations are commonly preferred, non-fasting concentrations have been used in research and shown to be useful in clinical decision making [33, 34]. Moreover, studies have shown that the measured lipids are only minimally affected by the fat composition in the diet [35, 36], and the non-fasting state was therefore accepted for the present study, as the purpose is to show the lipid profile in a naturalistic population. The HC participants were not fasting either and ate as usual. Samples were stored at − 80 °C until analysis at Denmark’s National Biobank at Statens Serum Institut, and afterwards analyzed at the National University Hospital, Rigshospitalet.

### Questionnaires

*Eating Disorder Examination (EDE-Q)*: The EDE-Q is a 28-item self-reported questionnaire derived from the Eating Disorder Examination (EDE) interview [37]. The EDE-Q has four subscales and a global score designed to assess eating disorder psychopathology. It concerns behaviors over a 28-day time period on the Restraint, Eating Concern, Shape Concern, and Weight Concern subscales. The global score is the sum of the four subscale scores divided by four (the number of subscales). Higher scores indicate greater levels of symptomatology.

*Eating Disorder Inventory (EDI-2)*: The EDI-2 is a self-report questionnaire for assessing the presence of behaviors and cognitions associated with eating disorders including AN (both restricting and binge-eating/purging subtypes), bulimia nervosa, eating disorder not otherwise specified including binge-eating disorder [38]. EDI-2 items are summed into 12 subscales: Drive for Thinness, Bulimia, Body Dissatisfaction, Low Self-Esteem, Personal Alienation, Interpersonal Insecurity, Interpersonal Alienation, Interoceptive Deficits, Emotional Dysregulation, Perfectionism, Ascetism, and Maturity Fears. The total score is the sum of the subscales.

*Major Depression Inventory (MDI)*: The MDI is a 10-item self-reported questionnaire used as a screening instrument for major depression and for measuring the severity of clinical depression [39]. Symptoms are assessed for the past 2 weeks, and a higher score signifies more severe depression. The score range is 0–50 with 0–20 indicating no or doubtful depression, 21–25 mild depression, 26–30 moderate depression, and 31–50 severe depression.

Furthermore, an in-house self-reported questionnaire was used to extract basic personal information, problems in relation to eating, socioeconomic status, and eating disorder history. Educational attainment was divided into four categories: short education (primary school and trained workers), secondary school (gymnasium), medium long (university college), long (university degree and doctorate).

### Statistics

Statistical analyses were performed on the open-source software R (version 3.6.3, Holding the Windsock, released on 2020-02-29, The R Foundation for Statistical Computing Platform: x86_64-w64-mingw32/×64) and STATA 17 (StataCorp LP, College Station, TX, USA).

Distribution was investigated, and due to skewness, non-parametric tests were used [40]. The differences between the AN and HC groups were assessed by the nonparametric test on the equality of medians, and the difference in the AN group before and after weight restoration treatment was assessed by Wilcoxon matched-pairs signed-rank test. Differences were considered statistically significant at *p* < 0.05. Due to the explorative nature of the study and suboptimal power of non-parametric statistical tests, the results were not adjusted for multiple tests. Furthermore, an adjustment for multiple tests would primarily affect the results in Table 5 i.e., the median regression analyses.

To analyze potential predictors of plasma lipid concentration, median regression analyses were carried out with pre-treatment plasma lipid concentrations (total cholesterol, HDL, LDL, VLDL, TG, and FFA) as the dependent variables, and age, BMI, MDI score, AN subtype (restrictive/binge-purge), illness duration, educational attainment, and illness severity as independent variables. Lower educational level has been linked to unfavorable lipid concentrations, and therefore, educational attainment was included as a potential predictor [41].

## Results

Characteristics of the AN sample are presented in Table 1. Almost two thirds of the sample had AN of the restricting type while the binge-eating/purging type comprised 37%. The most common comorbidities were depression and anxiety, diagnosed in about 40% of the sample (diagnostic information on comorbidities was extracted from the medical records). The median illness duration was 7 years (IQR 4.0–12.0), and of the 75 patients with AN, 90% were recruited from an inpatient ward. Median BMI increased from 15.3 (3.4) to 18.9 (2.9) kg/m^2^ during a median weight restoration treatment period of 63 days (IQR 35.0–90.5)*.*

**Table 1:** Characteristics of patients with anorexia nervosa

Variables	AN population	%
Anorexia nervosa subtype, N (%)		
Restricting type	47	62.7
Binge-eating/purging type	28	37.3
Remission status, N (%)		
Acute ill	75	100.0
Partial remission	–	–
Unit, N (%)		
Day hospital	< 5	–
Inpatient open ward	68	90.7
Intensive closed ward	< 5	–
Illness duration in years, median (IQR 25–75%)	7.0 (4.0–12.0)	
Weight restoration period in days, median (IQR 25–75%)	63 (35.0–90.5)	
Medication status, N (%)		
Antidepressive	30	40.0
Antipsychotics	23	30.7
Anxiolytics	10	13.3
Laxatives (prescribed)	14	18.7
Analgesic	23	30.7
Psychiatric comorbidities, N (%)		
Depression	14	18.7
Anxiety	16	21.3
Obsessive compulsive disorder (OCD)	< 5	–
Post-traumatic stress disorder (PTSD)	< 5	< 5
Bipolar disorder	< 5	< 5
Schizophrenia	< 5	< 5
Attention deficit hyperactivity disorder (ADHD)	< 5	< 5
Autism spectrum disorder (ASD)	< 5	< 5

AN = patients with anorexia nervosa, N = Number, IQR = interquartile range

< 5: values with a count of less than five have been suppressed in the table to protect participant confidentiality and comply with ethical guidelines

When we excluded the 23 patients (30.7%) who were taking antipsychotic medication (n = 52), results were similar to the full sample (n = 75) with significantly elevated plasma total cholesterol (*p* = 0.007) and plasma TG (*p* = 0.009) in patients with AN compared with HC. Additionally, plasma HDL concentration was significantly elevated in patients not taking antipsychotics, and FFA was significantly lower. Similar to the full sample, there were no changes in plasma lipid concentrations after weight restoration treatment. Therefore, we chose to use the full sample for further analyses. Additionally, when we excluded day hospital patients, results were also similar to the full sample, and therefore the full sample was used.

### Comparison of the AN and HC groups

Baseline characteristics for the AN and HC samples are presented in Table 2. Baseline BMI and weight were significantly lower for individuals with AN compared with HC. A significantly higher number of HC had a partner and the distribution of education differed significantly between the two groups with longer education in the HC group. Pre-treatment BMI, plasma lipid and sex hormone concentrations for the AN group are compared with the HC group in Table 3. Pre-treatment concentrations of plasma total cholesterol and TG were significantly higher in the AN group compared with the HC group, while estradiol, testosterone, and LH were significantly lower. Similar differences between the AN and HC groups were observed between the AN post-treatment levels and the HC group (*p* value for AN post-treatment compared with HC not shown). Table 4 shows substantial and highly significant differences between the pre-treatment scores of the AN group and the scores of the HC group on all EDE-Q and the EDI scales as well as the MDI depression scale.

**Table 2:** Characteristics of the study population at baseline (T_0_)

Variables	AN	%	HC	%	*p* value
N	75	–	26	–	–
Age, median (IQR)	24 (10)	–	28 (5.8)	–	0.7
BMI (kg/m^2^), median (IQR)	15.3 (3.3)	–	22.4 (2.9)	–	**< 0.001**
Weight (kg), median (IQR)	42.4 (8.1)	–	65 (15.3)	–	**< 0.001**
Height (cm)	167 (11.5)	–	170 (9)	–	0.05
Smokers, N (%)	19	25.0	< 5	–	0.4
Period during the past 6 months, N (%)	22	29.3	25	96.2	**< 0.001**
Educational attainment^a^, N (%)					
Short	22	29.3	0	–	**< 0.001**
Secondary school	34	45.3	9	34.6	0.4
Medium long	17	22.7	11	42.3	0.07
Long	< 5	–	6	23.1	**0.003**
Origin other than Danish, N (%)	7	9.3	3	11.5	1.0
Has children, N (%)	6	8.0	4	15.3	0.5
Partner, N (%)	17	22.7	15	57.7	**0.007**
Parental separation, N (%)	24	32.0	10	38.5	0.6
Parent deceased, N (%)	11	14.7	5	19.3	0.8

AN = patients with anorexia nervosa, HC = healthy control participants, N = Number, IQR = interquartile range, BMI = body mass index. *p* value: differences were assessed by the nonparametric test on the equality of medians. Bold font = signifcant results

^a^Educational attainment was divided into four categories: short education (primary school and trained workers), secondary school (gymnasium), medium long (university college), long (university degree and doctorate). The distribution of education differed significantly between the two groups with longer education in the HC group (χ2 = 21.0, *p* = 0.0001)

< 5: Values with a count of less than five have been suppressed in the table to protect participant confidentiality and comply with ethical guidelines

**Table 3:** Plasma concentrations of lipids and sex hormones

Variables	AN pre (T_0_)	AN post (T_1_)	Difference AN	*p* value AN pre versus AN post	HC	*p* value HC versus AN pre
N	75	75			26	
BMI (kg/m^2^)	15.3 (3.4)	18.9 (2.9)	2.4 (2.8)	**< 0.001**	22.4 (2.9)	**< 0.001**
Total cholesterol (mmol/L)	4.9 (1.2)	4.9 (1.4)	0.1 (1.0)	0.2	4.3 (0.7)	**0.003**
HDL (mmol/L)	2.0 (0.5)	1.9 (0.6)	− 0.03 (0.2)	0.4	1.7 (0.6)	0.06
LDL (mmol/L)	2.3 (1.0)	2.4 (1.0)	0.07 (0.8)	0.4	2.3 (0.9)	0.8
VLDL (mmol/L)	0.5 (0.3)	0.5 (0.3)	0.03 (0.4)	0.1	0.4 (0.2)	0.7
FFA (mmol/L)	0.1 (0.1)	0.1 (0.1)	0.01 (0.1)	0.4	0.2 (0.3)	0.1
TG (mmol/L)	1.2 (0.8)	1.1 (0.7)	− 0.04 (0.9)	0.6	0.9 (0.3)	**0.006**
Estradiol (nmol/L)	0.05 (0.1)	0.09 (0.1)	0.02 (0.1)	0.06	0.3 (0.3)	**< 0.001**
Testosterone (nmol/L)	0.5 (0.7)	0.4 (0.6)	0 (0.3)	0.7	0.8 (0.5)	**0.03**
Progesterone (nmol/L)	0.5 (0.5)	0.6 (0.4)	0.05 (0.5)	0.6	1.9 (27.6)	0.8
SHBG (nmol/L)	44.1 (32.1)	33.5 (21.1)	− 9.9 (18.3)	**< 0.001**	43.4 (14.7)	0.98
FSH (IU/L)	4.1 (5.3)	5.2 (3.3)	0.7 (3.4)	**0.03**	4.9 (2.3)	0.2
LH (IU/L)	1.2 (7.9)	4.5 (8.6)	1.5 (4.1)	**< 0.001**	6.8 (3.8)	**< 0.001**

All results are reported as median (IQR). IQR = interquartile range, AN pre = patients with anorexia nervosa, pre-treatment/at baseline (T_0_), AN post = patients with anorexia nervosa, post-treatment/at follow-up (T_1_), HC = healthy control participants, N = number, BMI = body mass index, HDL = high-density lipoprotein cholesterol, LDL = low density lipoprotein cholesterol, VLDL = very low density lipoprotein cholesterol, FFA = free fatty acids, TG = triglycerides, SHBG = sex-hormone binding globulin, FSH = follicle stimulating hormone, LH = luteinizing hormone

*p* value: differences were assessed by the nonparametric test on the equality of medians, and the difference in the AN group before and after weight restoration was assessed by Wilcoxon matched-pairs signed-rank test. Bold font = signifcant results

**Table 4:** Results from self-reported questionnaires

Variables	AN pre (T_0_)	AN post (T_1_)	Difference AN	*p* value AN pre versus AN post	HC	*p* value HC versus AN pre
EDE-Q, mean (IQR)						
Global score	4.17 (2.14)	4.2 (2,21)	0 (0)	1.0	0.54 (1.09)	**< 0.001**
Restraint subscale	3.1 (2.8)	3.4 (2.6)	0 (0)	0.8	0.4 (1)	**< 0.001**
Eating concern subscale	3.3 (2.2)	3.1 (2.2)	0 (0)	1.0	0 (0.4)	**< 0.001**
Shape concern subscale	5.06 (2.25)	5 (2.12)	0 (0)	1.0	0.75 (1.63)	**< 0.001**
Weight concern subscale	4.4 (2.6)	4.4 (2.6)	0 (0)	1.0	0.6 (1.6)	**< 0.001**
EDI, mean (IQR)						
Global score	170 (76)	165 (86)	0 (9)	1.0	38.5 (35)	**< 0.001**
Drive for thinness subscale	19 (14)	19 (13)	0 (1)	0.6	2.5 (5)	**< 0.001**
Bulimia subscale	3 (6)	3 (5)	0 (1)	0.4	1 (2)	**0.02**
Body dissatisfaction subscale	29 (13)	29 (14)	0 (1)	0.7	7 (15)	**< 0.001**
Low self-esteem subscale	15 (10)	16 (9)	0 (1)	0.1	2 (3)	**< 0.001**
Personal alienation subscale	14 (8)	14 (7)	0 (1)	1.0	2 (6)	**< 0.001**
Interpersonal insecurity subscale	13 (9)	12 (9)	0 (2)	0.9	3 (3)	**< 0.001**
Interpersonal alienation subscale	10 (7)	9 (8)	0 (1)	0.7	3 (5)	**< 0.001**
Interoceptive deficits subscale	19 (15)	18 (12)	0 (3)	0.1	2 (3)	**< 0.001**
Emotional dysregulation subscale	6 (6)	6 (6)	0 (0)	1.0	1 (3)	**< 0.001**
Perfectionism subscale	12 (7)	12 (8)	0 (1)	1.0	4 (3)	**< 0.001**
Ascetism subscale	11 (9)	9 (10)	0 (2)	0.7	1.5 (4)	**< 0.001**
Maturity fears subscale	11 (12)	11 (11)	0 (1)	0.1	5.5 (6)	**0.009**
MDI global score, mean (IQR)	33 (17)	30.5 (19)	0 (5)	**0.007**	6 (5)	**< 0.001**

All results are reported as median (IQR). IQR = interquartile range, AN pre = individuals with anorexia nervosa, pre-treatment/at baseline (T_0_), AN post = individuals with anorexia nervosa, post-treatment/at follow-up (T_1_), HC = healthy control participants, EDE-Q = eating disorder examination questionnaire, EDI = eating disorder inventory, MDI = major depression inventory

*p* value: differences were assessed by the non-parametric test on the equality of medians, and the difference in the AN group before and after weight restoration was assessed by Wilcoxon matched-pairs signed rank test. Bold font = significant results

### Change with weight restoration

Table 3 shows a significant increase in BMI after weight restoration treatment (median 18.9 kg/m^2^), but no significant changes in plasma concentrations of the lipids (total cholesterol and TG). In fact, the only significant change was observed in the sex hormone category, where SHBG showed a significant decrease and FSH and LH a significant increase. However, Table 4 shows no significant changes in EDI and EDE-Q subscales or global scores after weight restoration treatment; yet there was a relatively small, but significant decrease in MDI depression scores.

### Predictors of plasma lipid concentrations

In the AN group, pre-treatment total cholesterol (r = 0.29, *p* = 0.01), TG (r = 0.40, *p* = 0.0004), and VLDL (r = 0.34, *p* = 0.003) were significantly positively correlated with illness duration. However, in median regression models no significant predictors of HDL, VLDL, and FFA were identified (see Table 5). Illness duration was positively associated with only TG. Age was positively associated with both total cholesterol and LDL, whereas long education was positively associated with TG and negatively associated with LDL. Yet, Table 5 shows that most of these associations were only significant at the 0.05 level and would not be significant if multiple testing was considered.

**Table 5:** Median regression model for patients with anorexia nervosa at baseline (T_0_)

Analysis	Predictor	Regression coefficient	95% CI	*p* value
Total cholesterol	Age	0.06	0.01; 0.11	**0.02**
	BMI (kg/m^2^)	− 0.04	− 0.16; 0.08	0.5
	MDI (depression)	− 0.01	− 0.05; 0.02	0.5
	AN subtype	0.32	− 0.32; 0.95	0.3
	Illness duration	− 0.02	− 0.07; 0.04	0.6
	Short education	0.27	− 0.38; 0.92	0.4
	Medium-long education	− 0.02	− 0.96; 0.93	0.97
	Long education	− 0.33	− 2.11; 1.46	0.7
	EDI-2 total score	− 0.001	− 0.01; 0.01	0.8
	EDE-Q global score	0.24	− 0.04; 0.52	0.1
HDL	Age	0.01	− 0.01; 0.03	0.3
	BMI (kg/m^2^)	− 0.04	− 0.09; 0.01	0.1
	MDI (depression)	− 0.0002	− 0.01; 0.01	0.98
	AN subtype	− 0.10	− 0.37; 0.16	0.4
	Illness duration	− 0.009	− 0.03; 0.02	0.5
	Short education	− 0.07	− 0.34; 0.21	0.6
	Medium-long education	0.25	− 0.14; 0.64	0.2
	Long education	− 0.05	− 0.79; 0.70	0.9
	EDI-2 total score	− 0.001	− 0.004; 0.002	0.5
	EDE-Q global score	0.09	− 0.03; 0.20	0.1
LDL	Age	0.05	0.01; 0.10	**0.01**
	BMI (kg/m^2^)	− 0.004	− 0.10; 0.09	0.9
	MDI (depression)	− 0.01	− 0.04; 0.02	0.4
	AN subtype	− 0.01	− 0.54; 0.51	0.96
	Illness duration	− 0.04	− 0.08; 0.01	0.1
	Short education	0.005	− 0.53; 0.54	0.98
	Medium-long education	0.08	− 0.70; 0.86	0.8
	Long education	− 1.64	− 3.11; − 0.17	**0.03**
	EDI-2 total score	0.00002	− 0.01; 0.01	0.996
	EDE-Q global score	0.08	− 0.14; 0.31	0.5
VLDL	Age	0.003	− 0.01; 0.02	0.7
	BMI (kg/m^2^)	0.01	− 0.03; 0.04	0.7
	MDI (depression)	− 0.003	− 0.01; 0.01	0.5
	AN subtype	0.12	− 0.06; 0.31	0.2
	Illness duration	0.004	− 0.01; 0.02	0.7
	Short education	0.001	− 0.19; 0.19	0.99
	Medium-long education	0.11	− 0.18; 0.39	0.5
	Long education	0.2	− 0.34; 0.73	0.5
	EDI-2 total score	− 0.0002	− 0.002; 0.002	0.9
	EDE-Q global score	0.05	− 0.03; 0.13	0.2
TG	Age	0.001	− 0.02; 0.03	0.9
	BMI (kg/m^2^)	− 0.02	− 0.07; 0.04	0.6
	MDI (depression)	− 0.01	− 0.02; 0.01	0.5
	AN subtype	0.14	− 0.16; 0.45	0.4
	Illness duration	0.03	0.002; 0.06	**0.04**
	Short education	0.17	− 0.14; 0.48	0.3
	Medium-long education	0.21	− 0.24; 0.66	0.4
	Long education	1.0	0.17; 1.89	**0.02**
	EDI-2 total score	− 0.001	− 0.004; 0.003	0.7
	EDE-Q global score	0.09	− 0.04; 0.23	0.2
FFA	Age	− 0.001	− 0.004; 0.002	0.5
	BMI (kg/m^2^)	− 0.003	− 0.01; 0.004	0.4
	MDI (depression)	− 0.001	− 0.003; 0.001	0.5
	AN subtype	− 0.02	− 0.06; 0.02	0.3
	Illness duration	0.001	− 0.003; 0.004	0.8
	Short education	0.03	− 0.02; 0.07	0.2
	Medium-long education	0.03	− 0.03; 0.09	0.3
	Long education	− 0.04	− 0.16; 0.07	0.4
	EDI-2 total score	− 0.0002	− 0.0006; 0.0003	0.5
	EDE-Q global score	0.01	− 0.01; 0.03	0.4

Median regression. CI = confidence interval, BMI = body mass index, MDI = major depression inventory questionnaire, AN = anorexia nervosa, EDI-2 = eating disorder inventory 2, EDE-Q = eating disorder examination questionnaire, HDL = high density lipoprotein cholesterol, LDL = low density lipoprotein cholesterol, VLDL = very low density lipoprotein cholesterol, TG = triglycerides, FFA = free fatty acids. Bold font = significant results

## Discussion

The findings of the present study are based on data from a large, prospective cohort study including a case–control study at baseline comparing patients with AN with HC. Previous longitudinal studies investigating lipids in AN are few and inconsistent, and with the present study we confirmed that (1) plasma concentrations of lipids are significantly higher in individuals with AN compared with HC, and that (2) plasma lipid concentrations are persistently elevated with weight restoration. The median regression analyses suggested that age, illness duration, and long educational level are associated with plasma lipid concentrations.

### Lipids: comparison of the AN and HC groups

The observed significantly elevated total cholesterol concentrations are in accordance with a systematic review and meta-analysis [6]. Similarly, we also found significantly elevated TG in accordance with other studies [6, 9, 17].

### Lipids: change after weight restoration treatment

The concentration of total cholesterol and TG was higher in individuals with AN compared with HC, and these lipids remained elevated after partial weight restoration treatment despite significantly increased BMI in the same time period. Similarly, FFA, estradiol, testosterone, and progesterone were significantly lower compared with HC and remained so after partial weight restoration. While lipid concentrations were stable, a significant decrease in depressive symptoms after partial weight restoration was observed, but there was no significant change in self-reported eating disorder symptoms.

The studies included in the meta-analysis on lipid concentrations in patients with AN compared with HC, were heterogenous and included few longitudinal studies [6]. Consequently, the replication in the present study of significantly increased total cholesterol and TG, and of no significant change after weight restoration treatment, corroborate previous findings in a larger, homogenous sample with normalized weight at follow-up.

The findings in the present study, with no significant changes after weight restoration treatment, could be due to an underlying pathophysiology with increased lipids not merely being a consequence rapid weight loss caused by starvation, but potentially part of an underlying, premorbid illness specific mechanism i.e., a trait/biomarker of AN. However, further investigation, with longer-follow-up is required to firmly conclude whether lipid alterations are a state or trait effect of AN.

The non-significant change in eating disorder symptoms, as assessed by EDI-2 and EDE-Q, were not consistent with the literature [42], and underscores that weight restoration treatment is only one component of recovery from eating disorder symptoms [43]. A possible explanation for the observed persistently high levels of eating disorder symptoms in the present study could be explained by the long median illness duration of 7 years. Weight restoration treatment was associated with changes in depressive symptoms (MDI score decreased from 33 to 30.5); however, core eating disorder symptoms usually take longer to change and require other forms of interventions. The majority of participants were admitted to inpatient departments (95%) reflecting their illness severity i.e., the eating disorder behavior could not be managed by outpatient treatment alone. It is possible that there is a sub-group in the AN population (e.g., with severe comorbidities or high levels of emotional dysregulation) with more crystallized eating disorder core pathology, which may require more intensive interventions beyond standard inpatient eating disorder treatment.

### Predictors of lipid concentrations

Despite a significant bivariate correlation between total cholesterol and AN illness duration, median regression analyses showed only age as a predictor of total cholesterol. However, the regression analyses suggested a number of associations with LDL and TG: age and long education predicted LDL, and illness duration and long education predicted TG. Similarly, the meta-analysis showed a positive association with TG for mean illness duration [6]. A study found an association between treatment non-response and illness duration, and expectedly the likelihood of poor therapy response was increased for individuals with AN with higher eating disorder symptomatology [44]. Longer illness duration could be related to illness progression which, perhaps, could be linked to high cholesterol. In the present study 47 individuals had restrictive type AN (62.7%) and 28 individuals had binge-eating/purging type AN (37.3%). Based on the worse outcome in the binge-eating/purging subtype [45], a larger sample size of both AN subtypes would enable an in-depth investigation. A recent systematic review and meta-analysis [45] collected evidence on the transition from restrictive AN to binge-eating/purging AN, and reported several worse outcomes including longer duration of illness, higher prevalence of past traumatic experience, comorbid mental disorders, somatoform dissociation and, suicidality related to the binge-eating/purging AN subtype [46–48]. As AN has one of the highest mortalities of all psychiatric disorders, partly due to suicide [49, 50], it could also be relevant to further investigate if there are specific subgroups, e.g. the binge-eating/purging subtype, with higher prevalence of suicidal ideation and suicide in relation to altered lipid concentrations [19]. As high blood cholesterol concentrations could act by increasing serotonin activity, high concentrations could also be a protective factor against suicidality [19, 20]. However, the present study only included 28 patients with the binge-eating/purging subtype of AN, and a larger sample would be required for further investigation.

### Sex hormones

At baseline, the significantly lower plasma estradiol, testosterone, and LH concentrations in AN compared with HC are consistent with the literature [22]. Diverging from the literature, we did not observe a significant difference in plasma progesterone and FSH concentrations between AN and HC [22, 51].

After weight restoration treatment, plasma estradiol and testosterone are persistently decreased. Similarly, consistent with the literature [52, 53] we found plasma SHBG concentrations to be significantly increased in T_0_ and decreased in T_1_ despite no significant changes when compared with HC. Plasma FSH and LH concentrations significantly increased post-treatment.

Considering the female/male-ratio in AN [54, 55] it is relevant to further investigate the ovarian hormones in relation to lipids, as cholesterol is a precursor to estradiol, and AN in the hypoestrogenic state is differentially associated with increased cholesterol in premenarchal and postmenarcheal female individuals [23].

As low estradiol concentrations seem to play a role in AN pathology, estrogen replacement trials have been conducted. However, estrogen replacement therapy did not improve eating disorder symptoms, but only showed a reduction in trait anxiety [56]. High comorbidity is reported between AN and anxiety, which is also supported by our findings where 21% had a diagnosis of anxiety according to DSM-5. Animal models and human laboratory studies indicate that low estrogen impairs fear extinction, and low estradiol concentrations could, therefore, play a role in maintaining the fear of food and fat in recently weight restored patients with AN [57]. Furthermore, an on-going study is investigating estrogen-progestin combination as add-on to inpatient psychotherapy in AN [58]. Likewise, low testosterone concentrations could contribute to anxiety, depression, and eating disorder symptoms in female patients with AN [59]. However, a randomized placebo-controlled trial of low-dose testosterone did not improve symptoms [60]. However, estradiol/SHBG and testosterone/SHBG are used as indices for the bioactive hormone concentrations. Therefore, the decreased SHBG concentration at T_1_ could result in a relatively higher concentration of free plasma estradiol and testosterone, which could mean the increase is higher than represented in the total concentrations. For progesterone, there is still a lack of understanding of its role in AN, however, looking to other psychiatric disorders, progesterone concentrations seem to play a role in postpartum depression and premenstrual syndrome [61, 62].

### Limitations and strengths

We excluded all participants who took oral contraceptive pills, but there was no information about if/when participants stopped taking oral contraceptive pills. The participants with AN were assessed twice while the control participants were only assessed once. Due to the naturalistic study design, the participants (both individuals with AN and HC) were not fasting at the time of blood sample withdrawal, which could have affected plasma TG concentrations. Additionally, comparisons of dietary content (including supplement use) between the AN group during renutrition and the HC group was not analyzed and could be impacting data. The main strength of this study was the prospective design with a control group. Furthermore, the study was homogenous, with a large sample size, included comprehensive psychometric self-report instruments, and a longitudinal design with a substantial weight gain. Furthermore, baseline measurements were conducted after stabilization.

### Implications for future research

Future research can build on these results and investigate lipid metabolism in-depth focusing on each point of the lipid metabolism pathway to narrow down the point of alterations in patients with AN compared with HC. Our findings of persistently high plasma lipid concentrations together with recent findings on a possible increased risk of thromboembolism in patients with AN undergoing weight restoration treatment [63], could point towards an increased risk of cardiovascular risk factors which should be investigated further, along with the indication for lipid-lowering medication. Furthermore, weight restoration studies comparing different diets could be relevant in the light on a recent lipidomics study finding similarities between the lipidomes of refed patients with AN and patients with obesity, insulin resistance and type II diabetes [27].

## Conclusion

In this observational, prospective study we found significantly higher plasma lipid concentrations in a group of patients with AN than in HC. The lipid concentrations in the AN group did not change significantly in the AN group after weight restoration treatment, and in the sex hormone category only SHBG decreased significantly while FSH and LH increased. Furthermore, regression analyses showed illness duration to be a predictor for TG, age was a predictor for total cholesterol and LDL, while long education also predicted LDL and TG. Likewise, we found no significant improvement in eating disorder symptoms (measured by EDE-Q and EDI-2) after weight restoration treatment, although the depressive symptoms decreased. These findings could point towards an underlying pathophysiology with increased lipids not merely being a consequence of rapid weight loss caused by starvation, but perhaps part of an underlying illness mechanism i.e., a premorbid trait of AN. However, this is speculative, since we do not have pre-morbid measures, and therefore, the observed results could also be a “scar effect” rather than a pre-morbid trait persisting after recovery. Furthermore, considering the short-term follow up in the present study, it is not possible to say if the lipids normalize after a longer follow-up. The etiology and illness mechanism of AN remains uncertain, and our study is a small step in understanding the effects of starvation and underlying biology of patients with AN.

### What is already known on this subject?

Blood lipid concentrations have been reported to be elevated in low weight patients with anorexia nervosa (AN) compared with healthy controls (HC). However, studies longitudinally investigating blood lipid concentrations in AN before and after weight restoration treatment are sparse.

### What this study adds?

This large (n = 75) observational, prospective study found significantly higher plasma lipid concentrations in a group of patients with anorexia nervosa (AN) compared with healthy controls (HC). The lipid concentrations in the AN group did not change significantly after weight restoration treatment. There was also no significant improvement in eating disorder symptoms (measured by EDE-Q and EDI-2) after weight restoration treatment, although the depressive symptoms decreased. Regression analyses showed illness duration to be a predictor for triglycerides (TG), age was a predictor for total cholesterol and low-density lipoprotein (LDL), while long education also predicted LDL and TG. More longitudinal studies with longer follow-up time are needed to conclude if elevated plasma lipids are a consequence of rapid weight loss, part of underlying disease mechanism/premorbid trait or a result of a "scar effect".

## Data Availability

The datasets are not publicly available because informed consent provisions did not cover public data sharing. However, datasets are available from the corresponding author.

## Abbreviations

AN: Anorexia nervosa
BMI: Body mass index
HC: Healthy control participants
PROLED: PROspective Longitudinal all-comers inclusion study in Eating Disorders
ARFID: Avoidant/restrictive food intake disorder
DSM-5: The Diagnostic and Statistical Manual of Mental Disorders 5th edition
EDE: Eating Disorder Examination
SCID: Structured Clinical Interview for DSM Disorders
REDCap: Research Electronic Data Capture
HDL: High-density lipoprotein
LDL: Low-density lipoprotein
VLDL: Very low-density lipoprotein
TG: Triglycerides
FFA: Free fatty acids
SHBG: Sex-hormone binding globulin
LH: Luteinizing hormone
FSH: Follicle-stimulating hormone
ECLIA: Electrochemiluminescence immunoassay
EDE-Q: Eating Disorder Examination
EDI-2: Eating Disorder Inventory
MDI: Major Depression Inventory
